# Breaking the glial loop: astrocytic PAD2 and citrullinated vimentin drive microglial dysfunction in Alzheimer’s disease

**DOI:** 10.1186/s13024-026-00974-w

**Published:** 2026-07-13

**Authors:** Jeff Y. L. Lam, Guojun Bu

**Affiliations:** Division of Life Science and State Key Laboratory of Nervous System Disorders, The Hong Kong University of Science and Technology, Hong Kong, China

**Keywords:** Alzheimer’s disease, Astrocytes, Microglia, Glial crosstalk, Protein post-translational modification, Vimentin

## Abstract

Zhang et al. reveal that astrocytic PAD2-mediated citrullination of vimentin drives a TLR4-dependent pro-inflammatory loop in microglia, linking glial crosstalk to impaired amyloid clearance and identifying a potential therapeutic and biomarker pathway in Alzheimer’s disease.

## Main text

In Alzheimer’s disease (AD), interactions between astrocytes and microglia in response to amyloid-β (Aβ) plaques contribute to a chronic inflammatory environment that promotes synaptic dysfunction and neurodegeneration [1–2]. Although this bidirectional communication is well established, the molecular mediators that connect these glial populations remain incompletely defined. In a recent study published in *Immunity *[1], Zhang et al. identify a mechanism involving protein post-translational modifications that mediates this process. They show that astrocytic peptidyl-arginine deiminase 2 (PAD2) catalyzes the citrullination of vimentin (Cit-vimentin), which is subsequently released and acts on microglia via Toll-like receptor 4 (TLR4), promoting pro-inflammatory activation and impairing Aβ phagocytosis. Suppression of this pathway reduces amyloid pathology and improves cognitive function in AD models, suggesting its potential as a therapeutic target.

Historically, AD research has focused on the direct toxicity of amyloid and tau, resulting in the development of disease-modifying therapies targeting Aβ and, more recently, tau pathology. Monoclonal antibody-based approaches have demonstrated that reducing amyloid burden can modestly slow cognitive decline, supporting Aβ as a tractable therapeutic target [3]. Importantly, emerging evidence suggests that the effectiveness of immunotherapies depends on the antibody engagement of microglia, whose activation promotes Aβ clearance through coordinated functional reprogramming [11]. However, the overall efficacy of these strategies remains limited, which highlights the need to better understand complementary disease mechanisms, including neuroinflammation. Accumulating genetic and multi-omics evidence further supports the central roles of glial cells in AD pathogenesis [4–6], driven by both their cell-autonomous mechanisms and non-cell-autonomous crosstalk. Microglia and astrocytes surrounding Aβ plaques form a reactive niche in which microglial signals induce astrocytic reactivity, while astrocytes reciprocally modulate microglial activation and survival. Despite this conceptual framework, the molecular determinants that sustain this feedback loop remain poorly defined.

Zhang et al. address the molecular basis of this feedback loop by examining protein citrullination [1], an irreversible post-translational modification mediated by PAD enzymes [5–6]. Using human postmortem brain samples and amyloid mouse models (5xFAD and APP/PS1), they demonstrate that PAD2 expression and global citrullination are increased in plaque-associated astrocytes. Conditional deletion of *Padi2* in astrocytes (*Padi2*^f/f^; *Aldh1l1*-CreER^T2^) in 5xFAD mice reduces Aβ deposition, improves cognitive performance, and shifts microglia toward a homeostatic phenotype, supporting a causal role for astrocytic PAD2 [1].

To further address the mechanism, the authors identify vimentin as a major substrate of PAD2 in reactive astrocytes. Increased intracellular calcium in early disease stages enhances PAD2 activity, leading to site-specific citrullination of vimentin at residues 175 and 184. Cit-vimentin is released into the extracellular space, where it functions as a ligand for microglial TLR4 [7]. This interaction activates NF-κB signaling and suppresses oxidative phosphorylation, thereby reducing microglial capacity for Aβ clearance [8]. Genetic ablation of *Tlr4* in microglia abolishes the effects of extracellular citrullinated vimentin, confirming its role in mediating astrocyte-microglia communication [1].

These findings have important therapeutic implications. Broad anti-inflammatory approaches in AD have shown limited efficacy, likely due to non-specific effects on immune functions. In contrast, targeting astrocytic PAD2 may selectively disrupt a pathogenic signaling axis. Consistent with this idea, pharmacological inhibition of PAD2 via intracerebroventricularly delivery of the small molecule AFM30a recapitulates the effects of genetic deletion, including normalization of astrocytic and microglial profiles, restoration of synaptic markers, and improved cognitive performance in AD mice [1].

In addition, Cit-vimentin may serve as a biomarker. Elevated levels are detected in cerebrospinal fluid and plasma in AD models, and correlate with plaque burden and glial activation [1, 9]. This raises the possibility that circulating Cit-vimentin could be used to monitor disease progression or therapeutic response, although validation in human cohorts is required.

Several questions remain. Beyond citrullination, vimentin undergoes other post-translational modifications, including truncation, which promotes apoptosis and impairs astrocytic function in amyloid transport and clearance [10]. The multifactorial roles of vimentin, the precise sequence of these events, and their downstream functional impacts warrant further studies. Although vimentin is a key PAD2 substrate, other citrullinated proteins may also contribute to additional aspects of AD pathology. Furthermore, the specificity and sensitivity of Cit-vimentin as a biomarker for AD and other neurodegenerative conditions will need to be established in large and diverse cohorts. In addition, the therapeutic applicability of AFM30a requires further evaluation given its invasive route of administration and the necessity for repeated high dosing. While AFM30a demonstrates robust overall efficacy, more refined PAD2 inhibitors such as the blood-brain barrier-penetrant variants currently under development by the authors may be needed to optimize outcomes, alongside careful consideration of the therapeutic window during AD progression. Finally, the mechanisms underlying the selective restoration of postsynaptic, but not presynaptic, terminals following PAD2 inhibition remain to be fully elucidated.

In summary, Zhang et al. define a PAD2-Cit-vimentin-TLR4 signaling axis that mediates pathological astrocyte-microglia interactions in AD^1^. This work provides mechanistic insight into glial crosstalk and identifies a potential target for therapeutic intervention.

## Data Availability

No datasets were generated or analysed during the current study.

## Abbreviations

AD: Alzheimer’s disease
Aβ: Amyloid-β
PAD2: Peptidyl-arginine deiminase 2
Cit-vimentin: Citrullination of vimentin
TLR4: Toll-like receptor 4
